# Correction to “Succinate Dehydrogenase Pathogenic Variants Among Older Adults With Head and Neck Paragangliomas”

**DOI:** 10.1002/lio2.70347

**Published:** 2026-01-22

N. Bellamkonda, A. Naumer, L. O. Buchmann, et al., “Succinate Dehydrogenase Pathogenic Variants Among Older Adults With Head and Neck Paragangliomas,” *Laryngoscope Investigative Otolaryngology* 10, no. 6 (2025): e70302, https://doi.org/10.1002/lio2.70302.

In the last sentence of paragraph two of the Results section, the text—“Among the 21 patients with a HNPGL‐associated PV, two were between 50 and 59 years of age, seven between 60 and 69, eight between 70 and 79, three between 80 and 89, and one between 90 and 99 (Figure 1)”—was incorrect.

It should have read: “Among the 21 patients with a HNPGL‐associated PV, ten were diagnosed with their first HNPGL between 50 and 59 years of age, ten between 60 and 69, and one between 70 and 79 (Figure 1).”

Figure 1, which corresponds directly to this text, has been updated with the following figure.

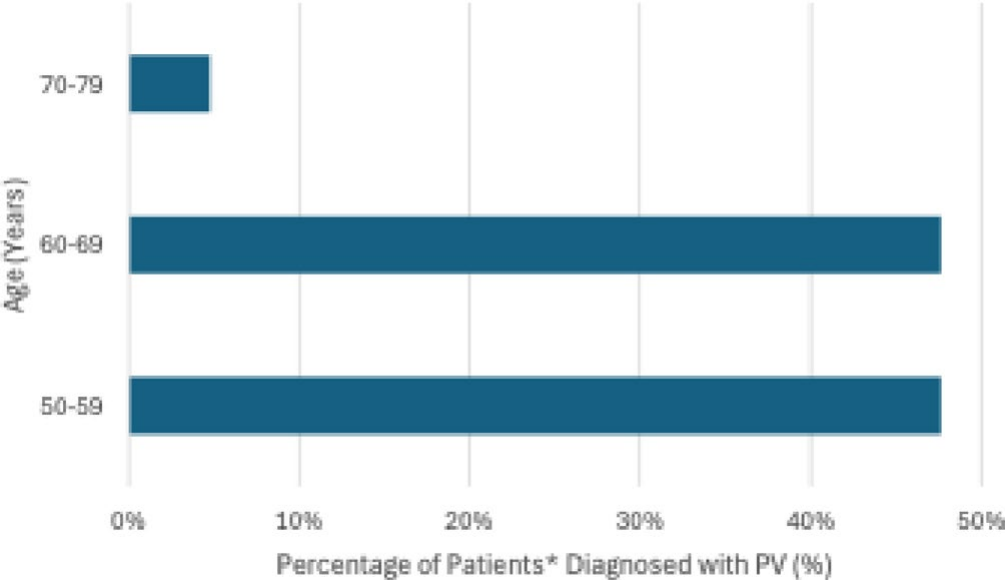

We apologize for this error.

